# Warburg Effects in Cancer and Normal Proliferating Cells: Two Tales of the Same Name

**DOI:** 10.1016/j.gpb.2018.12.006

**Published:** 2019-05-07

**Authors:** Huiyan Sun, Liang Chen, Sha Cao, Yanchun Liang, Ying Xu

**Affiliations:** 1 The China-Japan Union Hospital, Jilin University, Changchun 130033, China; 2 MOE Key Laboratory of Symbolic Computation and Knowledge Engineering, College of Computer Science and Technology, Jilin University, Changchun 130012, China; 3 Computational Systems Biology Lab, Department of Biochemistry and Molecular Biology and Institute of Bioinformatics, University of Georgia, Athens, GA 30602, USA; 4 Faculty of Health Sciences, University of Macau, Taipa, Macau SAR 999078, China; 5 Department of Biostatistics, School of Medicine, Indiana University, Indianapolis, IN 46202, USA; 6 Zhuhai Laboratory of MOE Key Laboratory of Symbolic Computation and Knowledge Engineering, Zhuhai College of Jilin University, Zhuhai 519041, China

**Keywords:** Cancer, Warburg effect, Fenton reaction, Cell proliferation, pH homeostasis

## Abstract

It has been observed that both **cancer** tissue cells and normal proliferating cells (NPCs) have the **Warburg effect**. Our goal here is to demonstrate that they do this for different reasons. To accomplish this, we have analyzed the transcriptomic data of over 7000 cancer and control tissues of 14 cancer types in TCGA and data of five NPC types in GEO. Our analyses reveal that NPCs accumulate large quantities of ATPs produced by the respiration process before starting the Warburg effect, to raise the intracellular pH from ∼6.8 to ∼7.2 and to prepare for cell division energetically. Once cell cycle starts, the cells start to rely on glycolysis for ATP generation followed by ATP hydrolysis and lactic acid release, to maintain the elevated intracellular pH as needed by cell division since together the three processes are pH neutral. The cells go back to the normal respiration-based ATP production once the cell division phase ends. In comparison, cancer cells have reached their intracellular pH at ∼7.4 from top down as multiple acid-loading transporters are up-regulated and most acid-extruding ones except for lactic acid exporters are repressed. Cancer cells use continuous glycolysis for ATP production as way to acidify the intracellular space since the lactic acid secretion is decoupled from glycolysis-based ATP generation and is pH balanced by increased expressions of acid-loading transporters. Co-expression analyses suggest that lactic acid secretion is regulated by external, non-pH related signals. Overall, our data strongly suggest that the two cell types have the Warburg effect for very different reasons.

## Introduction

Otto Warburg published his seminal paper in 1927 on the observation that cancer cells tend to allocate substantial fractions of glucose to glycolytic ATP production followed by lactate generation rather than by the TCA cycle and the respiration chain regardless of the O_2_ level, which is referred to as the Warburg effect [1] and serves as the basis for PET/CT based cancer detection. This observation has perplexed generations of cancer researchers, since the respiration pathway is considerably more efficient for ATP generation than glycolysis, with the former producing 36 ATPs and the latter producing 2 ATPs per glucose. Multiple hypotheses have been put forward about why cancer cells do this as follows. (1) Cancer cells may have dysfunctional mitochondria, which was later proved to be incorrect [2], [3], [4]. (2) The glycolytic pathway is faster than the respiration pathway for synthesizing the same number of ATPs from glucose, hence selected to support the rapid cell proliferation in cancer [5]; but this view is challenged as ATP is shown to be not a rate-limiting factor in cancer proliferation [6]. (3) Cancer cells have reduced mitochondrial activities due to hypoxia, partially because of the increased generation of reactive oxygen species (ROS) [7] and nitric oxide production [8]; however, cancer cells are known to have respiration activities across different cancer types and high levels of such activities are reported in some cancers [9]. (4) Cancer cells are lack of NAD^+^, hence using the conversion of pyruvate to lactate for NAD^+^ production [5], which was argued against since most of the relevant carbons were excreted, hence there is no net NAD^+^ production [10]. And (5) Warburg effect is a common characteristic of all proliferating cells, including cancer cells and NPCs [5]. While other hypotheses are largely considered as unacceptable [11], the last one has gained popularity in the past few years [12], [13], which we address here.

We have approached this problem from the perspective of intracellular acid-base homeostasis. It is known that healthy human epithelial cells have a mildly acidic intracellular pH at ∼6.8 and a basic extracellular pH at ∼7.2, while cancer cells have reversed these pH levels with intracellular pH at 7.2–7.4 [14] and extracellular pH at 6.6–6.8 [15]. To understand how cancer cells have reversed the intracellular and extracellular pH levels, we have examined the gene expression levels of all H^+^, OH^−^ and HCO_3_^−^ related plasma-membrane transporters across 14 cancer types, which include all the cancer types in the TCGA database with sufficiently large numbers (at least 10) of cancer tissues. To our surprise, we note that all cancer cells consistently up-regulate the expressions of multiple acid-loading transporters and repress the expressions of most acid-extruding transporters except for the lactic acid exporters throughout the progression of the cancers examined. This strongly suggests that there must be some unidentified metabolic processes that continuously produce alkaline molecules, since that the gradient-driven lactic acid exporters cannot accomplish the reversal between the intracellular and extracellular pH levels, at least not by the transporters alone.

We have recently predicted [16], through mining cancer tissue transcriptomic data and mathematical modeling, that cancer cells have Fenton reactions: F_e_^2+^ + H_2_O_2_ → F_e_^3+^ + ·OH + OH^−^ in their cytosol. Fenton reactions result from local iron accumulation and elevated H_2_O_2_ concentration due to increased local populations of innate immune cells, specifically neutrophil and macrophages. We predict that cancer cells of all the 14 cancer types examined have such reactions persistently using superoxide ($O_{2}^{∙-}$), produced by local innate immune cells and the host cells’ mitochondria, as the key reducing element of F_e_^3+^. We have further shown that OH^−^ produced by such reactions would ultimately overwhelm the cytosolic pH buffer, and hence drive up the intracellular pH. As response, various processes are induced to acidify the intracellular space to maintain the acid-base homeostasis [16].

We have predicted that glycolytic ATP synthesis represents a key responding process for acidifying the intracellular pH, since glycolytic ATP synthesis is pH neutral, while respiration-based ATP production consumes one proton per ATP and hydrolysis of any ATP releases one proton [17]. That is, synthesis of each glycolytic ATP releases one net proton when it is consumed while a respiration-synthesized ATP will be neutral when the ATP is hydrolyzed. In addition, it is also known that the level of glycolysis correlates strongly with the level of the predicted cytosolic Fenton reaction [16].

In contrast, NPCs generate ATPs through the respiration pathway and accumulate ATPs before cell proliferation. NPCs will partially switch to glycolytic ATP production when sufficiently large numbers of ATPs are intracellularly accumulated, hence driving up the intracellular pH as well as preparing for cell division energetically. During proliferation, consumption of an ATP leads to the release of a proton, coupled with the lactate generated from pyruvate at the end of glycolysis. Therefore, serving two purposes, that is, to maintain the raised pH level needed for proliferation and replenish each consumed ATP.

## Results

### Warburg effects in cancer *vs*. in activated NPC samples

We have used the following criteria to determine if a cancer tissue (and a NPC sample) has the Warburg effect: (i) Expressions of the genes encoding lactate dehydrogenase unit A or B, *LDHA* or *LDHB*, and of the genes encoding the main lactic acid exporters, *SLC16A1* or *SLC16A3*, are considerably up-regulated (fold change >2) in cancer tissue (and activated NPC cells) in comparison to the controls; and (ii) the proportion of the glycolytic flux via pyruvate kinase (PK, encoded by *PKM*) into the TCA cycle via pyruvate dehydrogenase (PDH, encoded by *PDHB*) decreases in cancer tissues (and activated NPC cells) in comparison to controls. Here, we use the normalized expression of *PDHB* against that of *PKM* as an approximation to the fraction of the metabolic efflux out of PK into the TCA cycle via PDH.

**Supplementary Figure S1:**
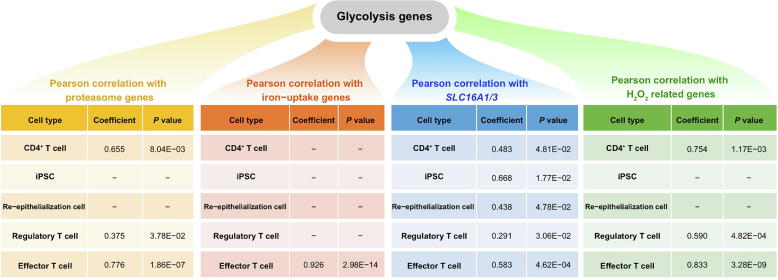
**Boxplots represent gene expression levels of Warburg effect related genes across different stages of the 13 cancer types** Gene expression levels of Warburg effect related genes *SLC16A1*/3, *LDHA*, *PKM*, and *PDHB* against *PKM*, across 13 cancer types: n is for control, s1 is for stage 1, s2 is for stage 2, s3 is for stage 3 and s4 is for stage 4 from left to right. The stage information of prostate cancer in TCGA is not available, hence prostate cancer is not included in the analysis.

**Supplementary Figure S2:**
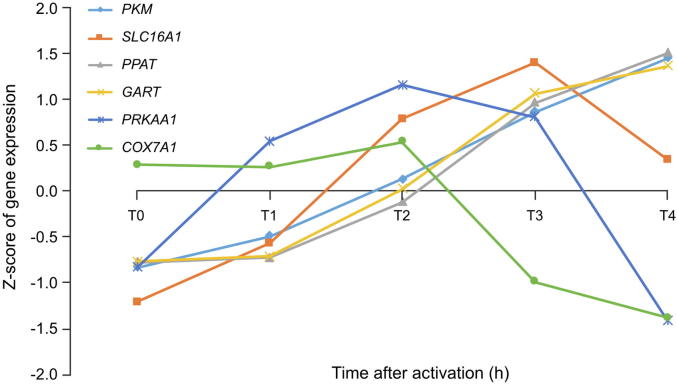
**Boxplots represent gene expression levels of Warburg effect related genes in five NPC: control and activated** Gene expression levels of Warburg effect related genes *SLC16A1*/3, *LDHA*, *PKM*, and *PDHB* against *PKM* across five NPC in control and activated.

We note that the expression of *LDHA* is up-regulated in the cancer tissues of all 14 cancer types examined except for LIHC (Figure S1). In addition, the expression of either *SLC16A1* or *SLC16A3* is up-regulated in the cancer tissues of all 14 cancer types except for COAD (see Materials and Methods for definition), which is known to have weak Warburg effect and hence generally not detected via PET/CT. In addition, the relative ratio of *PDHB/PKM* decreases in cancer tissues across different stages in comparison to the controls. Similarly, we found increased expression of *SLC16A1* and *LDHA* (or *LDHB*) in all the five NPC types (Figure S2). However, the relative *PDHB/PKM* ratio is reduced only in CD4^+^ T cells and effector T cells but remains comparable in the other three cell types in comparison to the matched controls. Hence, we predict that all the cancer types and the NPCs under consideration have the Warburg effect.

### Opposite behaviors of pH-related transporters in cancer *vs.* NPCs

**Figure 1:**
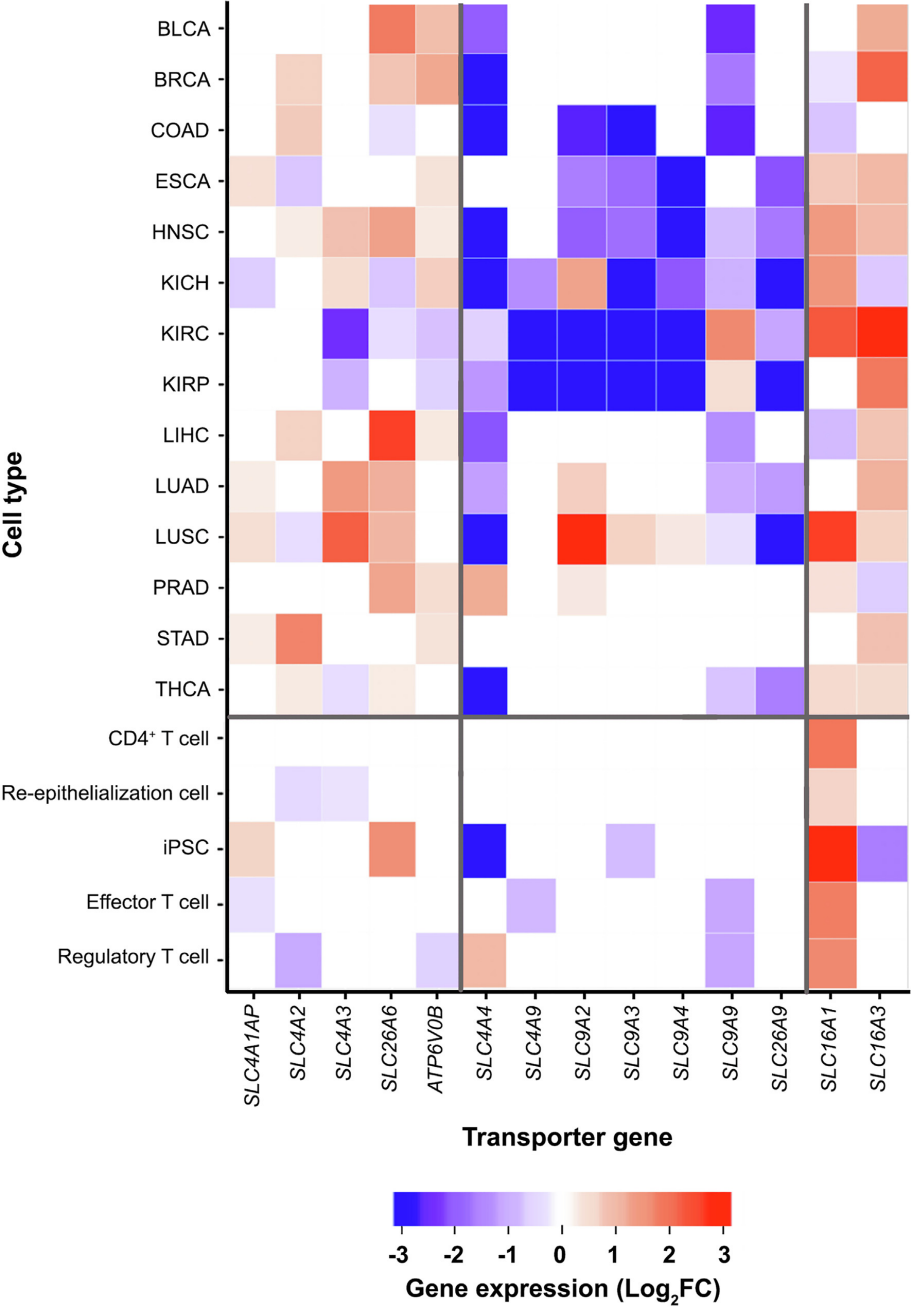
**Heatmaps for differential expressions of the selected transporter genes** The first zone is for acid-loading transporters, the second for acid-extruding transporters, and the last for lactate acid extruding transporters. Gene expression levels are indicated with FPKM presented as log_2_FC, with log_2_FC > 0.6 for up-regulated genes and log_2_FC < −0.6 for down-regulated genes. FC, fold change.

We have examined the gene expression levels of all the selected pH-related plasma-membrane transporters in cancer and in NPCs (see Materials and methods). We now go through the key differences between the expression patterns of these genes in groups in cancer *vs*. NPCs, with the detailed comparisons given in Figure 1. Note that in the following, the first three groups of genes are acid-loading transporter genes, and the next four are acid-extruding ones.

#### Acid-loading transporter genes

Among the acid-loading transporter genes, the expressions of *SLC4A1AP*, *SLC4A*2, and *SLC4A*3 are up-regulated or remain unaltered in cancer tissues *vs*. controls across most of the 14 cancer types. In comparison, the expressions of these genes are mostly down-regulated or remain unchanged across all the NPCs except for *SLC4A1AP*, which is up-regulated in iPSC.

The expression of *SLC26A6* is up-regulated or remains the same in 11 of the 14 cancer types except for COAD, KIRC, and KIRP. In comparison, the expression of *SLC26A6* is not changed in the activated NPC samples *vs.* controls except for the iPSC samples.

For the ATP6V genes, we have considered the expressions of *ATP6V0B* and *ATP6V0C* since their protein products are known to be localized in plasma membrane and have the same expression by definition. Table S1 shows the subcellular locations for the *ATP6V0* protein products predicted by Genecards [18], and Table S2 gives the predicted *ATP6V0B* expressions in both cancers and NPCs. We can see from the table that the gene is up-regulated across all cancer types except for KIRC and KIRP; and it has no change or is down-regulated in its expressions across all the NPC samples in the activated state *vs.* the control.

#### Acid-extruding transporter genes

Among the four groups of acid-extruding transporter genes, *SLC4A4*/*9* are down-regulated or show no changes in their expressions across all cancer types except for PRAD with *SLC4A4* being up-regulated. In comparison, these genes show no changes or are up-regulated in their expressions in all the activated NPCs *vs.* the controls except for iPSC and effector T cell, in which *SLC4A4* and *SLC4A9* are down-regulated, respectively.

For *SLC9A2*, *3*, *4*, *9*, they are mostly down-regulated in cancer *vs.* controls, mostly by *SLC9A9* and *SLC9A2*, while majority of these genes show no changes in their expressions in the activated NPCs *vs.* controls.

*SLC26A9* is predominantly down-regulated in cancer, and has no change in NPCs.

The only group of genes considered here has the same behaviors between cancer *vs.* NPCs is *SLC16A1*/3, with their expressions up-regulated in both cancer and the activated NPCs.

In sum, expressions of the acid-loading transporter genes are largely up-regulated, whereas expressions of the acid-extruding transporter genes are down-regulated in cancer *vs.* controls except for *SLC16A1*/3. For the acid-loading transporter genes, NPCs generally show the opposite gene-expression patterns to those in cancer while the acid-extruding transporters mostly show no changes in their expressions. Hence we conclude that cancer cells use the transporters under consideration to acidify their intracellular pH while NPCs alkalinize it. Given that both cancer and NPCs have comparable intracellular pH level [14], [19], we infer that cancer cells reach their pH level from above while NPCs get there from below.

A natural question would be: how can cancer cells maintain a basic intracellular pH when they are continuously acidified by powerful transporters? First, we posit that *SLC16A1*/3 are not the reason since they are driven by proton gradients, hence impossible for them to reverse the intracellular and extracellular pH. More importantly, there are numerous up-regulated acid-loading transporters with comparable expression levels (Figure 1), and multiple such transporters have higher Vmax values than SLC16A1/3. For instance, the Vmax of V-ATPase is 3–4 orders of magnitude higher (∼40 nmol/min/mg) [20] than that of SLC16A1/3 (∼63.0 pmol/min/mg) [21]. These data indicate that the acidification rate is substantially higher than the alkalinization rate by these transporters.

Actually cancer tissue cells are known to utilize a number of other metabolic processes to acidify their intracellular space. These include: (i) diffusion of fatty acids from blood circulation into cancer cells, hence acidifying the intracellular space since their pKa values are approximately 4.5 [22], lower than the intracellular pH; (ii) diffusion of NH_3_ out of cancer cells into blood circulation [23], thus making the intracellular pH more acidic, knowing that its pKa is 9.26 [22]; (iii) biosynthesis and deployment of large quantities of sialic acids and gangliosides [24], making the intracellular pH more acidic [25]; and (iv) glycolytic ATP production (see the next section).

All these data strongly suggest one possibility that there are unknown metabolic processes that continuously produce alkaline molecules inside cancer cells, which may have triggered all the above processes to continuously acidify the intracellular space to keep the cells viable.

### Fenton reactions in cancer cells and their impact on intracellular pH

We have recently build a computational model based on gene expression data of cancer *vs.* control tissues of all the 14 cancer types examined in this study, to demonstrate that all cancer tissue cells have Fenton reactions in their cytosol [16]. We outline the general idea of the study, for readers’ convenience.

It has been widely observed that cancer tends to be associated with chronic inflammation [26], which will give rise to elevated H_2_O_2_ level [27]. In addition, it has also been widely noted that cancer tends to have local accumulation of iron [28]. The combination of the two will result in an inorganic chemical reaction, called Fenton reaction: F_e_^2+^ + H_2_O_2_ → F_e_^3+^ + ·OH + OH^−^. Multiple authors have reported the observation of Fenton reactions in cancer across numerous cancer types [28], [29]. In our previous study, we have predicted that cancer cells generally use superoxide ($O_{2}^{∙-}$) as the reducing molecule to convert F_e_^3+^ to F_e_^2+^, produced predominantly by local innate immune cells including neutrophil and macrophages [16]. This will lead to persistent Fenton reaction, which can be rewritten as

$$O_{2}^{∙-}+H_{2}O_{2}\to ∙OH+{OH}^{-}+O_{2}$$

(also known as Haber-Weiss reaction) with Fe^2+^ as the catalyst and not consumed, where Fe^2+^ could be in iron-sulfur clusters or labile-iron pool [30]. Essentially, the reaction continuously produce ∙OH and ${OH}^{-}$, fueled by $O_{2}^{∙-}$ and $H_{2}O_{2}$ generated predominantly by local immune cells and catalyzed intracellular Fe^2+^.

Knowing that ∙OH can only be produced intracellularly by Fenton reactions when cells are not exposed to radiation [31], we have predicted if a cell harbors Fenton reaction in its cytosol if the quantities on two sides of the above chemical reaction strongly correlate with each other, given the level of Fe^2+^, specifically between [$∙OH]\;vs.\;\left[H_{2}O_{2}\right],\;\left[O_{2}^{∙-}\right]\;and\;\left[{Fe}^{2+}\right]$, where [X] represents the quantity of X. The rationale is that (1) we have observed that each of these quantities can be reliably estimated using the expressions of selected genes; and (2) the level of correlation between the two sides is highly consistent with the level of Fenton reaction [16]. File S1 and Table S3 show the statistical correlation between the two sides for all the cancer tissues of the 14 cancer types considered in our study [16]. Based on the data, we predict that all the cancer tissues have Fenton reactions in their cytosol.

In addition, we have also shown that Fenton reaction-produced OH^−^ can overwhelm the cytosolic pH buffer within a relatively short amount of time [16]. Then a range of processes is triggered to acidify the intracellular space, including the transporters under study, thus keeping its pH from becoming too high, since changes in intracellular pH can alter the whole biochemistry in a fundamental manner. In comparison, we predict that the activated NPCs have no or slight Fenton reactions based on data given in Table S4.

### Increased glycolytic ATP production is a response to cytosolic Fenton reactions

To pin down the possible reasons for the observed behaviors of the transporters in the first Results section, we searched for genes whose expressions correlate positively with those of the acid-loading transporter genes and simultaneously negatively with those of the acid-extruding ones for each cancer type. Table S5 lists the pathways enriched by such genes for each cancer type, with the detailed enrichment procedure given in File S1. Functional analyses reveal that these pathways largely fall into four categories: (1) cell proliferation and development; (2) macromolecular damages and degradation; (3) immune activities; and (4) stress response. These results point to the possibility that the behaviors of the transporters might be relevant to cytosolic Fenton reactions since as we previously shown [16] and outlined in the previous section that (i) Fenton reactions are the results of immune response to persistent irritations at the disease sites; and (ii) cytosolic Fenton reactions damage intracellular macromolecules by their ∙OH, and drive cell division by the persistent nucleotide synthesis induced as response to continuous production of OH^−^ [16].

**Figure 2:**
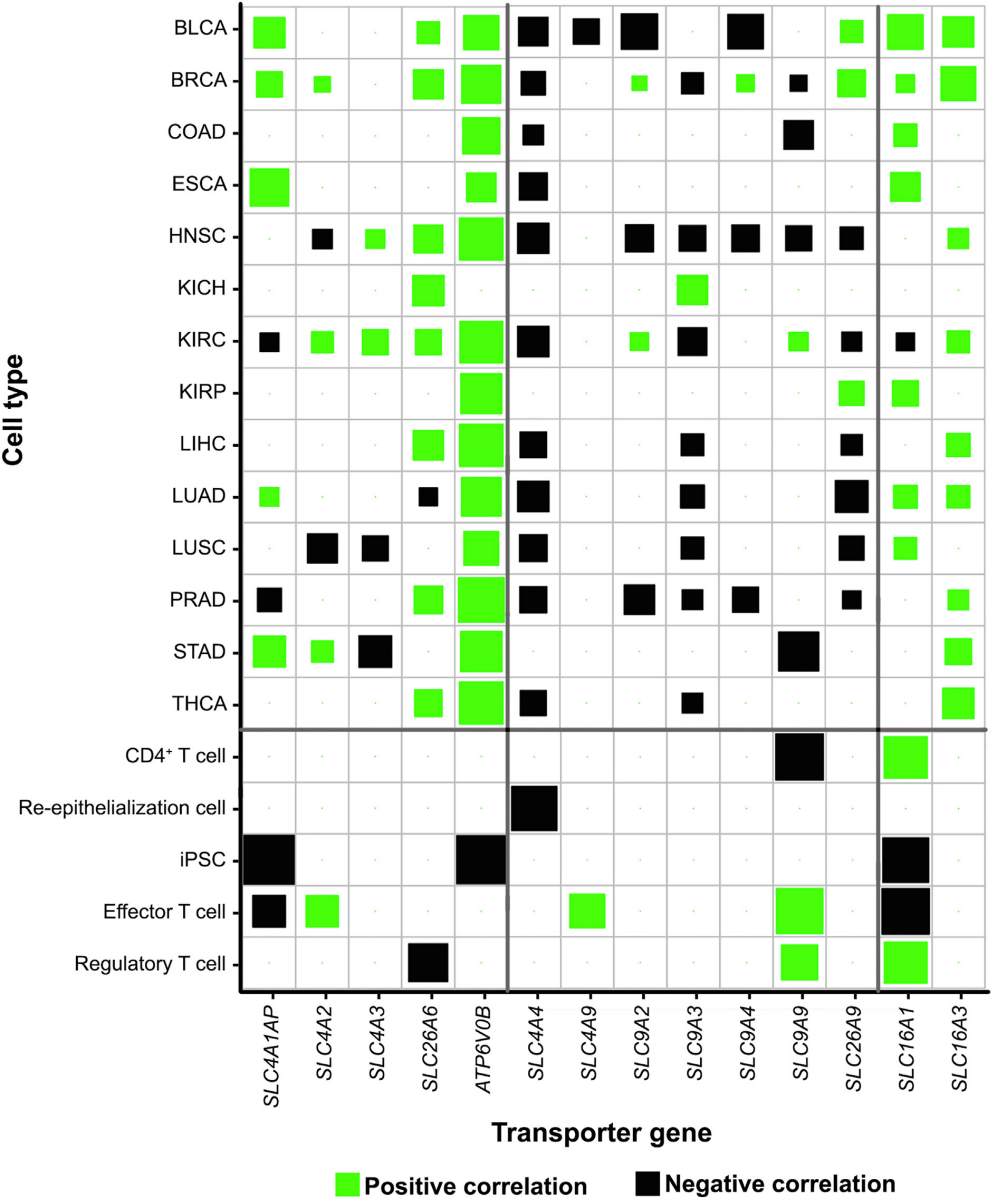
**Co-expression of proteasome (PSM) genes and acid-loading or acid-extruding transporter genes in 14 cancer types and NPCs** Co-expression was measured using Pearson correlation coefficient. The positive and negative correlations are indicated in green and black, respectively, with the size of each square representing the correlation level. Proteasome genes is given in Table S6.

Furthermore analyses have revealed that these transporter genes indeed strongly correlate genes/pathways used to define cytosolic Fenton reactions as detailed in Table S6 and Figure 2. Specifically, as shown in Figure 2, the expressions of proteasome (PSM) genes, one of the three gene groups used to define Fenton reaction (see later discussion of this section) positively correlate with those of the acid-loading transporter genes and negatively correlate with those of the acid-extruding transporter genes across all 14 cancer types. By these and the above paragraph, we predict the behaviors of these transporters are related to the OH^−^ produced by Fenton reactions. In contrast, the above correlations do not exist for NPC samples, as detailed in Table S7.

**Figure 3:**
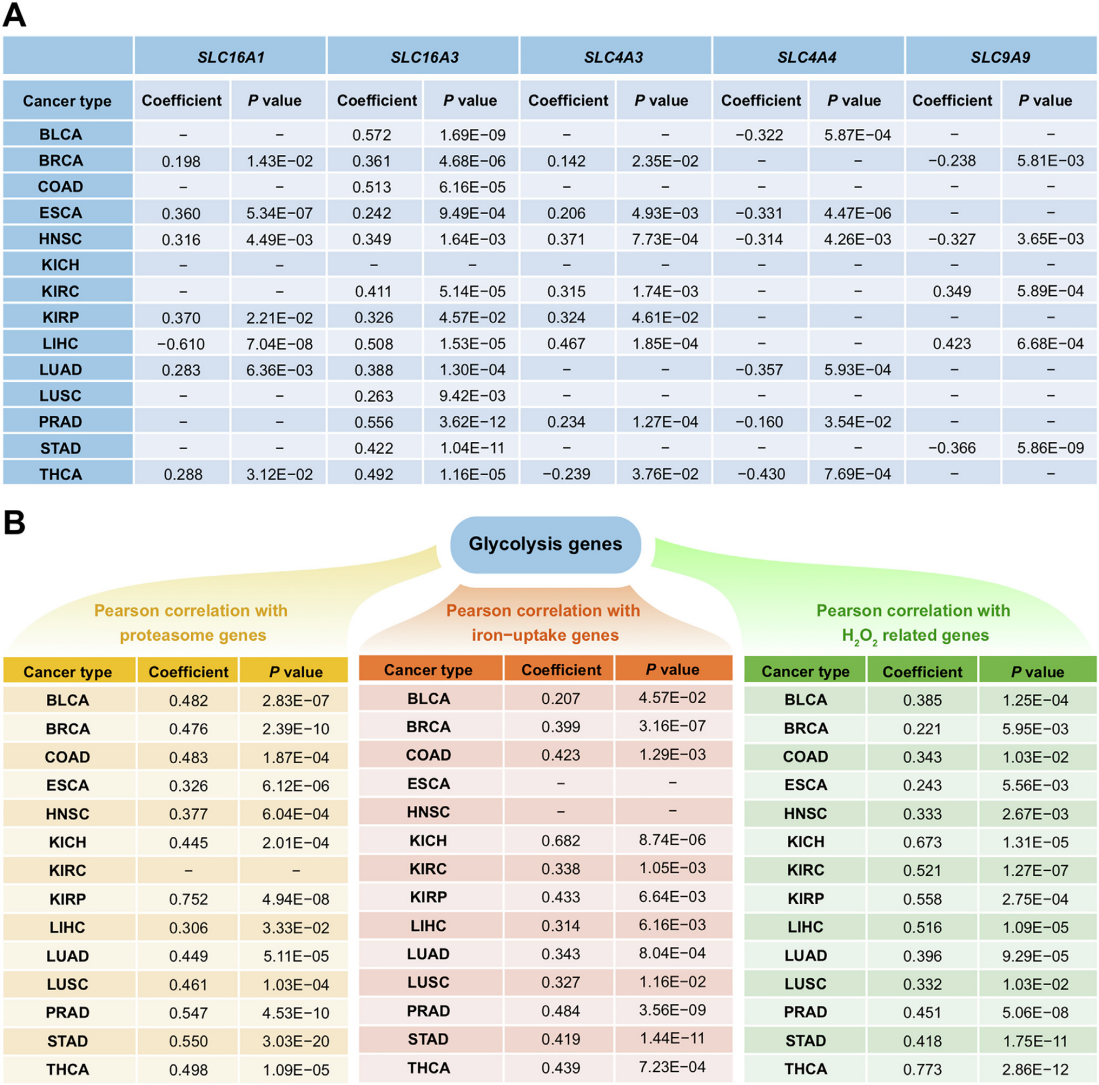
**Correlations between Fenton reaction level and ATP production genes** **A.** Correlation between *PKM* and one acid-loading and two acid-extruding transporter genes: *SLC4A3*, *SLC4A4*, and *SLC9A9*, *SLC16A1*, and *SLC16A3*. “−” represents the lack of the significant correlation. **B.** Pearson correlation between glycolytic ATP production genes (*PKM* and *PGK1*) and genes reflecting the levels of cytosolic Fenton reactions (proteasome genes, iron uptake genes and H_2_O_2_ related genes in Table S6) in cancer.

**Supplementary Figure S3:**
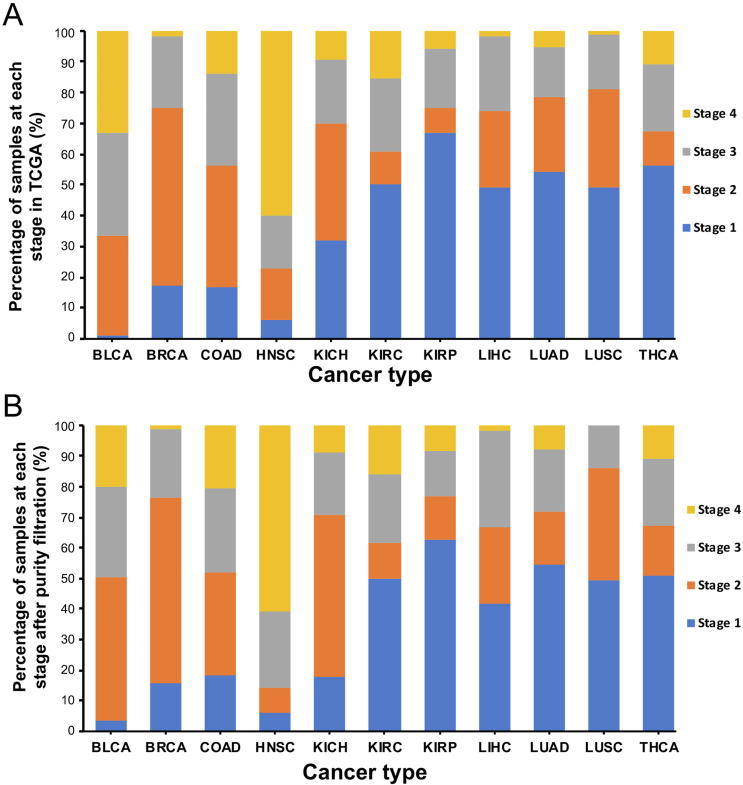
**Pearson correlations between *PKM* and genes used in Figure 3 in five NPCs** (1) Proteasome genes, (2) iron uptake genes, (3) *SLC16A1*/3, and (4) H_2_O_2_ related genes.

Interestingly, the correlation between *PKM* and *SLC16A1* in cancers is considerably weaker than it in NPCs as shown in Figure 3A and Figure S3, hence suggesting that the role of *SLC16A1* might be different from the other pH-related transporters.

We now show statistically that glycolytic ATP production is also relevant to cytosolic Fenton reactions. To accomplish this, we have calculated correlations between the level of glycolytic ATP production as reflected by the expression of *PKM* and (i) the level of protein damage as reflected by the expression of the *PSM* genes, (ii) the level of iron uptake represented by the expressions of *TFRC* (transferrin receptor) and *TFR2*, and (iii) the intracellular H_2_O_2_ level reflected by the expressions of *TXN* (thioredoxin), *TXN2*, *GCLC* (glutamate-cysteine ligase catalyst) and *GCLM*, respectively, with detailed results shown in Figure 3B. Note that the three groups of genes (i–iii) are used for establishing cytosolic Fenton reactions [16]. Hence, we conclude that the level of glycolytic ATP production strongly correlates with the level of Fenton reaction.

To see how glycolytic ATP production may be relevant to cytosolic pH, we note that the production of an ATP by respiration: ADP^3−^ + HPO_4_^2−^ → ATP^4−^ + OH^−^ consumes one proton, while ATP generation by glycolysis: glucose + 2ADP^3−^ + 2HPO_4_^2−^ → 2 lactate + 2 ATP^4−^ is pH neutral [17]. And hydrolysis of any ATP: ATP^4−^ + H_2_O → ADP^3−^ + HPO_4_^2−^ + H^+^ releases one proton. Hence, we conclude that glycolytic ATP production generates one net H^+^ for ATP when the ATP is hydrolyzed while in comparison, respiration based ATP production is pH neutral when the ATP is consumed.

By integrating all the above results, we predict that glycolytic ATP biosynthesis is a cellular response to the persistent OH^−^ production by cytosolic Fenton reactions across all 14 cancer types.

**Figure 4:**
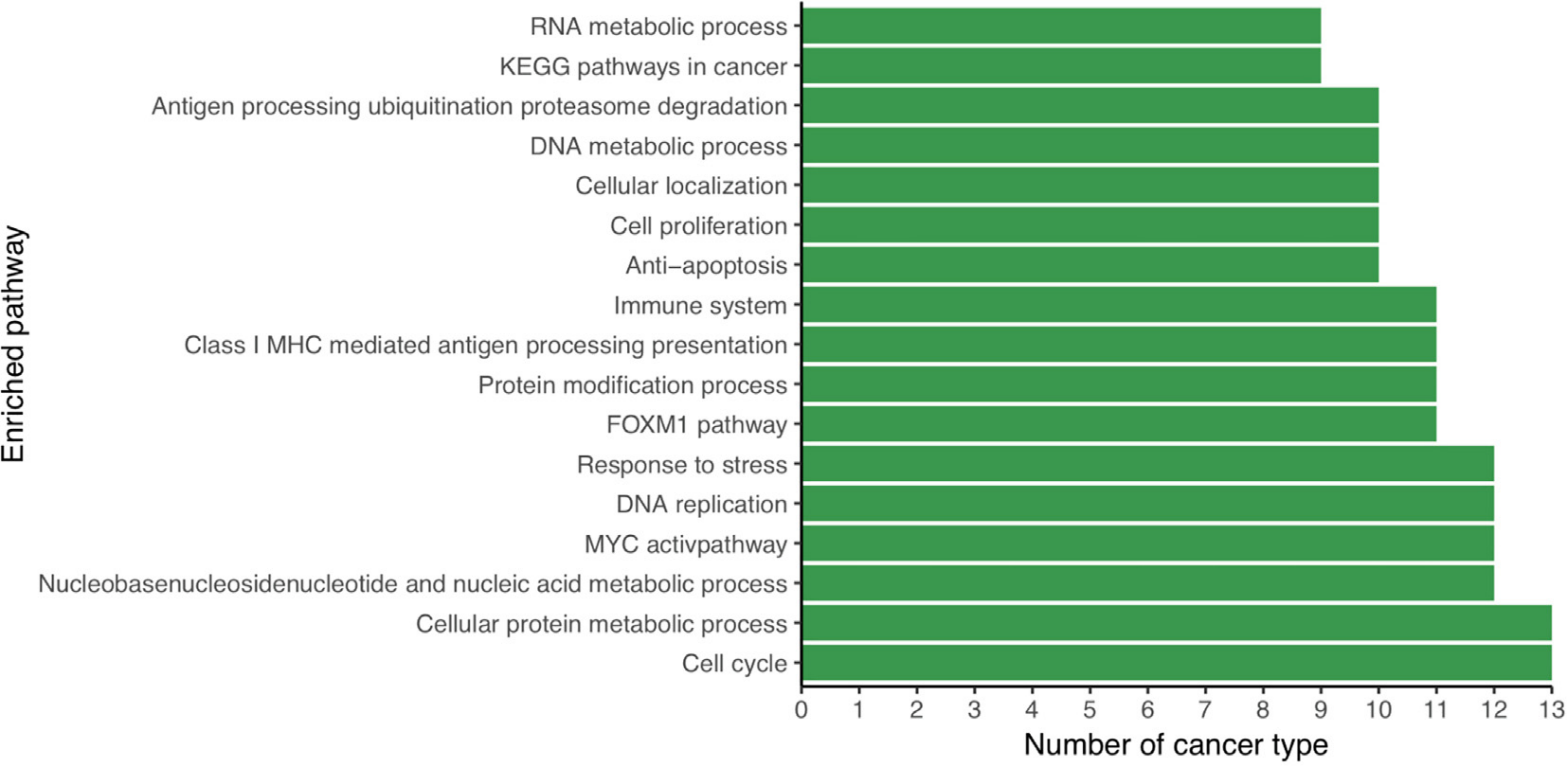
**The most commonly enriched pathways by genes strongly correlated with *SLC16A1* across the 14 cancer types** The length of a bar represents the number of cancer types where the pathway is enriched with genes whose expression strongly correlates with the expression of *SLC16A1*.

One puzzling issue remains: why do cancer cells secrete lactic acids (lactate + proton) when they face a major challenge to keep the intracellular pH from becoming too alkaline to remain viable? To address this issue, we have conducted correlation analyses between the expression levels of *SLC16A1* and all the up-regulated genes in each cancer type, followed by pathway-enrichment analyses of these genes. We have then examined the 100 most enriched pathways in each cancer type. We find that the most enriched pathways are involved in the biological processes related to immune system, cell cycle, and response to stress (Figure 4), hence suggesting that the secretion of the lactic acid might be regulated by external signals. This observation is consistent with previous studies suggesting that expression of *SLC16A1* is regulated by hypoxia [32], hyaluronic acid receptor *CD44* [33], and local stromal cells [33] in cancer.

Previous studies have suggested that lactic acids might serve protective roles in cancer against attacks by T cells [34]. Therefore, we conclude that lactic acid secretion by *SLC16A1* is most likely not related to intracellular pH homeostasis, instead it serves a protective role for cancer.

### Glycolytic ATP synthesis maintains the elevated intracellular pH during NPC proliferation

To understand the functional roles of glycolytic ATP production followed by lactic acid secretion in NPCs, we have performed correlation analyses between the expressions of *PKM* and genes related to the predicted cytosolic Fenton reaction on the NPC data. The analyses revealed that unlike cancer cells, no or very little correlation between expressions of glycolytic ATP production (*PKM* gene) and the Fenton reaction-defining genes, namely proteasome, iron uptake, and H_2_O_2_ genes in NPCs, as shown in Figure S3, hence suggesting that the reason for glycolytic ATP production in NPCs is different from that in cancers.

To probe why NPCs utilize glycolysis to produce ATP during their proliferating phase, we first review how unicellular organisms such as *Escherichia coli* and yeast control their cell cycle. It has been well established that in *E. coli*, nutrients are first used towards ATP production via the respiration process. This process switches largely to nucleotide and nucleotide-sugar syntheses once the cellular ATP concentration rises to a certain level, as result of that ATP production rate is higher than that of ATP consumption. Clearly, this will lead to increased cellular concentrations of nucleotides and nucleotide-sugar. It has been established that the cellular nucleotide-sugar concentration serves as the cue for cell cycle progression in *E. coli* [35] and *Bacillus subtilis* [36]. Hence when the cell cycle starts, the cells already have substantial levels of ATP accumulated needed for cell division.

The accumulation of the respiration-synthesized ATP also leads to an increase in the intracellular pH, as needed for the proliferation phase [37]. The reason is that respiration-based ATP biosynthesis consumes one proton per ATP, hence driving the pH up when the ATPs are accumulated. Since cell proliferation requires an elevated intracellular pH (from 6.8 to 7.2–7.4 [38]), cells must alter its way of ATP synthesis as otherwise the consumption of each ATP will release one H^+^, hence decreasing the pH. We predict that this is the reason that NPCs switch to glycolytic ATP production when cell proliferation starts. Details follow. Recall that the synthesis of each glycolytic ATP is pH neutral and produces on lactate [17]. When the respiration based ATP is consumed for cell proliferation, one H^+^ is generated. Now cells release this proton along with the lactate in the form of lactic acid. This serves two purposes: (1) maintaining the intracellular pH and (2) replenishing the consumed ATP. Again, it is worthy reemphasizing that the proton released along with lactate is NOT from glycolysis, instead, from hydrolysis of an ATP previously generated by respiration.

While this has not been demonstrated for normal human proliferating cells, we hypothesize that they basically follow a similar process to maintain a pH level needed for cell proliferation through glycolytic ATP production followed by lactic acid secretion. To provide supporting evidence, we have conducted a co-expression analysis between *SLC16A1* and all the up-regulated genes in each set of NPC samples, followed by pathway enrichment analyses. We find that majority of the enriched pathways are growth or development related. Moreover, over two thirds of the pathways that are most commonly *shared* by different NPCs are also growth or development related (Table S8), hence suggesting our prediction that the cellular roles of *SLC16A1* is different in cancer and in NPCs.

While we do not have experimental data to directly support the prediction that increased intracellular pH is essential to human cell proliferation, there are data that indirectly support our prediction. Specifically, we have analyzed a gene expression dataset (GSE77239) that was generated in an study aimed to examine the effect of inhibiting an acid-extruding pump in endothelial cells [39]. We have found that the expression levels of cell proliferation and glycolytic genes were reduced when the cells were treated by the inhibitor of the pump (Table S9).

**Figure 5:**
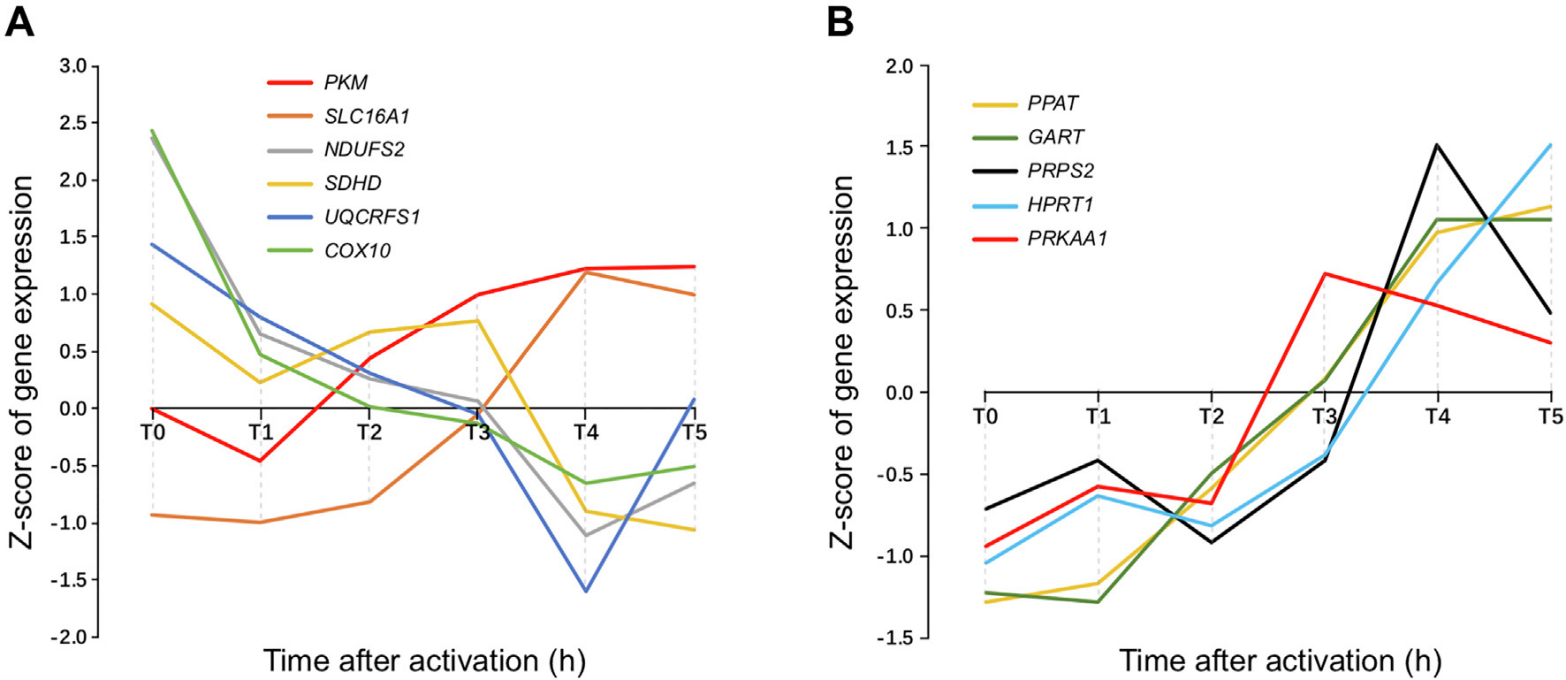
**Time-specific expression data of key genes involved in Warburg effect, electron transport chain and nucleotide synthesis pathways in GSE11292** The left panel shows the expression levels of *PKM*, *SLC16A1*, *NDUFS2*, *SDHD*, *UQCRFS1*, and *COX10*, four representative genes for electron transport chain Complex I, II, III and IV, respectively, where the time course data with 18 time points are grouped into six segments with each containing three consecutive points and *T_i_* represents the *i*th segment, 0 ≤ *i* ≤ 5. The right panel shows the expression levels of *PPAT*, *GART*, *PRPS2*, and *HPRT1* (blue), key genes in nucleotide synthesis pathways, as well as the expression level of *PRKAA*.

We have also studied the time-course data in one of the NPC datasets, GSE11292 for regulatory T cells and effector T cells. The dataset contains gene-expression data of the cells collected every 20 min at 19 time points starting from time zero. We have specifically examined genes involved in the respiratory chain, glycolysis, lactic acid secretion, and the gene, *PRKAA1*, involved in AMP degradation, whose expression level is known to be proportional to the intracellular ATP level [40], as shown in Figure 5. We find that the expression levels of the respiratory chain genes decrease continuously while the expressions of glycolytic ATP synthesis genes increase. In the meantime, the intracellular ATP concentration with the expression of *PRKAA1* as a readout reaches and stays at a high level and then gradually goes down starting at time T3 This observation is consistent with our model, namely, that (i) cell proliferation starts at a point when the intracellular ATP concentration reaches a high level; and (ii) the cells increase glycolytic ATP production and reduces respiration-based ATP synthesis during proliferation. Highly similar patterns are observed in other similar cases, as detailed in Figure S4.

It is noteworthy that unlike *E. coli*, respiratory ATPs are generated in mitochondria rather than in cytosol. Since cytosolic proton movement into mitochondria via the ATP synthase (or UCP transporters) is driven by proton gradient [41], we postulate that the increased mitochondrial pH would lead to increased cytosolic pH.

To estimate the number of respiratory ATPs that need to be accumulated to raise the intracellular pH from 6.8 to 7.4 (pH value needed for cell proliferation) in a normal human cell, we have calculated the number of protons needed to make such a change. Here we assume that the volume of the cell is 100 μm^3^, based on human cell data [42]. For the intracellular pH to change from 6.8 to 7.4, the concentration of the H^+^ needs to change from 10^−6.8^ to 10^−7.4^. Assuming that the pH buffering coefficient of the cell is ${2\times 10}^{5}$ for this pH range [43], the number of protons needed to make such a change is calculated as,

$$\left(10^{-6.8}-10^{-7.4}\right)\times 100\times {{2\times 10}^{5}\times 10}^{-15}\times 6.02\times 10^{23}≅1.43\times 10^{9}$$

where $6.02\times 10^{23}$ is the Asogadro constant. Hence, it takes approximately ${1.43\times 10}^{9}$ protons, hence this number of ATPs to make the desired pH change. Knowing that there are $\;6\times 10^{9}$nucleotides in human genome and it takes approximately five ATPs to synthesize one nucleotide on average, we predict that a cell must accumulate at least the number of ATPs needed to synthesize ∼4.7% (143/3000) of a human DNA to raise the pH from 6.8 to 7.4.

**Figure 6:**
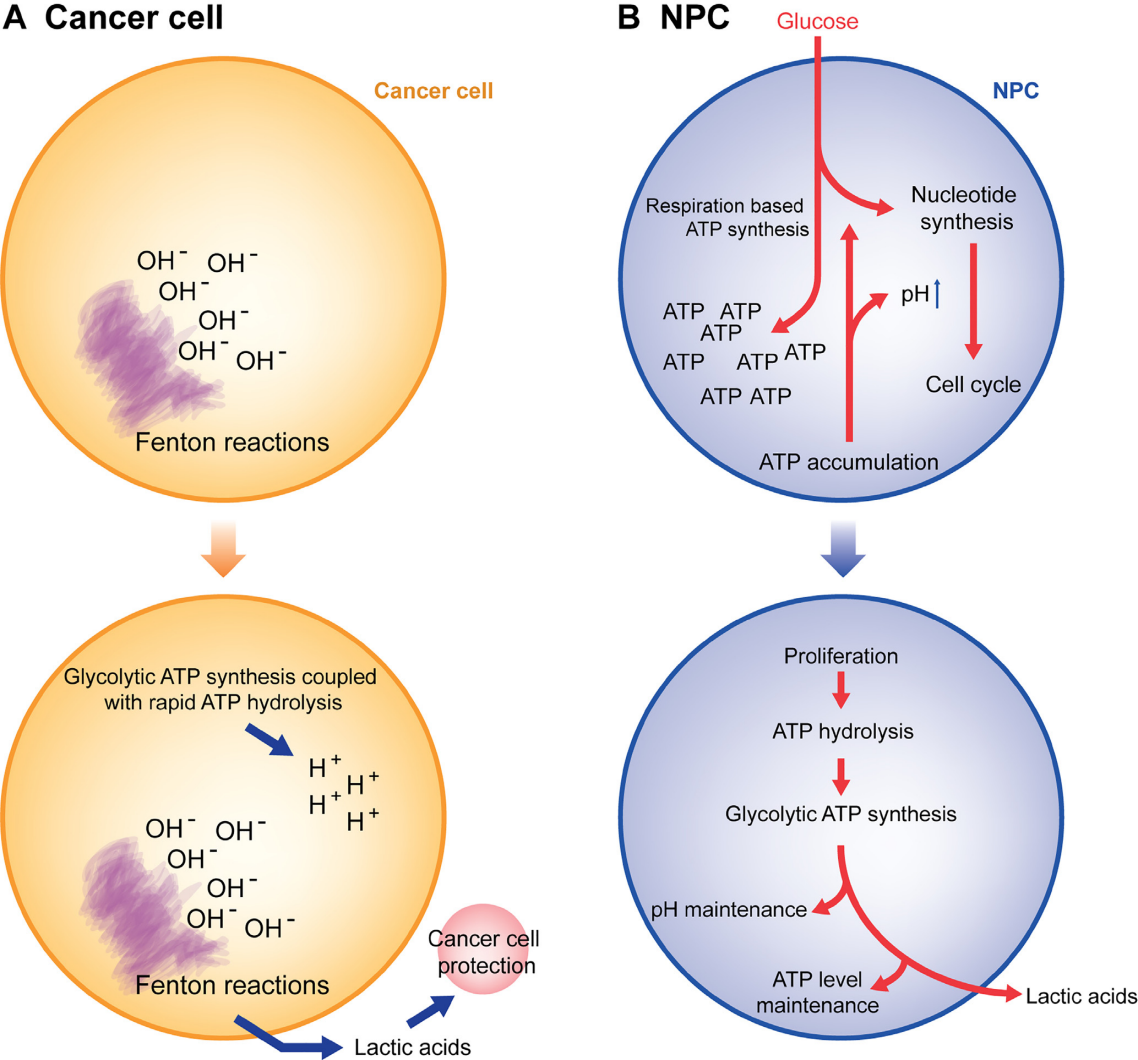
**Schematic illustration of the key differences between the Warburg effects in cancer cells *vs.* NPCs** **A.** Warburg effects in cancer. **B.** Warburg effects in normal proliferating cells.

Figure 6 summarizes the key differences between the Warburg effects in cancer cells *vs.* NPCs. Based on all the analyses, we predict that while both normal proliferating cells and cancer cells have the Warburg effect, they do it for fundamentally different reasons.

## Discussion

Several papers suggest that the Warburg effect is a common characteristic of all proliferating cells, including cancer and normal proliferating cells. Our comparative analyses of gene expression data between cancer tissue cells and NPCs provide strong evidence that they do this for fundamentally different reasons. Specifically, cancer cells do this mainly to produce net protons for neutralizing OH^−^ that is generated persistently by cytosolic Fenton reactions, whereas NPCs do this to maintain the elevated cytosolic pH needed for the optimal performance of the ribosomal proteins [37]. Moreover, cancer cells secrete lactic acids largely independent of lactate generation and they do this probably for protecting cancer cells from destruction by immune cells.

To avoid possible noises introduced by non-cancerous cells, we have selected in our analyses cancer tissue samples that are predicted to contain cancer cells with high purity. However, the results derived using such samples are essentially the same with the results derived from all samples of the 14 caner types in TCGA without this selection.

While our analyses provide generally consistent results across the 14 cancer types, we notice that kidney cancers tend to show different characteristics in maintaining their cytosolic acid-base homeostasis from other cancers, suggesting that further studies are needed.

We have also examined the protein abundances of the up-regulated genes in our model in the relevant cancer types (when available) from the Human Protein Atlas, and found that virtually all the highly up-regulated genes also have significant increase in protein abundance in the same cancer types if such data are available, hence indicating that our gene-expression based analyses are as valid as protein abundance based analyses if they were available.

Overall, the discovery made in this study offers a novel angle to examine cancer from the perspective of acid-base homeostasis. Our unique way in connecting coarse-grained information derived from gene-expression data with detailed chemistry properties of cells such as pH may offer a novel approach to tackle complex diseases like cancer, hence potentially leading to deeper understanding about cancer formation and development.

## Materials and methods

### Data processing and normalization

We have used RNA-seq data of 1612 samples covering 14 cancer types from the TCGA database [44], each having at least 13 cancer and (not necessarily paired) control samples, with gene expression levels indicated using normalized FPKM (with log2 transformation).

The purity of cancer tissue samples was predicted using five programs, namely ESTIMATE, ABSOLUTE, LUMP, IHC, and CPE [45]. Cancer tissue samples with the highest purity for each cancer type were selected, based on consensus results by the five methods. The detailed information about how tissue purity is assessed is given in File S1.

In addition, 143 samples of five NPC types with microarray-based gene expression data were selected from the GEO [46]. These include activated CD4^+^ T cells (GSE60235, GPL570) [47], induced pluripotent stem cell (GSE25970, GPL3921) [48], re-epithelialization cells (GSE28914, GPL570) [49], activated regulatory T cells (GSE11292, GPL570), and activated effector T cell (GSE11292) [50]. Each dataset consists of gene-expression data for both activated and inactive cell, used as controls. These cells are good representatives among proliferating noncancerous human cells, with the related datasets representing good qualities among all the relevant datasets in GEO. For a gene with multiple probes, we chose the probe having the highest average expression level to represent its expression. MAS5 in the R package “affy” [51] was used to normalize the gene expression data.

Table 1 summarizes the sample information for both cancer and NPC data used in this study. The original information of the cancer samples collected from TCGA before selection is given in Table S10 and Figure S5.

**Table 1:** **Samples of cancer tissues of 14 types and of five NPC types**

**Type**	**No. of cancer/activated samples**	**No. of control samples**
Bladder urothelial carcinoma (BLCA)	94	19
Breast invasive carcinoma (BRCA)	153	113
Colon adenocarcinoma (COAD)	55	41
Esophageal carcinoma (ESCA)	184*	13
Head and Neck squamous cell carcinoma (HNSC)	79	44
Kidney chromophobe (KICH)	34	25
Kidney renal clear cell carcinoma (KIRC)	91	72
Kidney renal papillary cell carcinoma (KIRP)	38	32
Liver hepatocellular carcinoma (LIHC)	65	50
Lung adenocarcinoma (LUAD)	92	59
Lung squamous cell carcinoma (LUSC)	59	51
Prostate adenocarcinoma (PRAD)	133	52
Stomach adenocarcinoma (STAD)	238*	33
Thyroid carcinoma (THCA)	56	59
Activated CD4 + T cells (GSE60235)	15	15
Activated effector T cells (GSE11292)	32	6
Activated regulatory T cells (GSE11292)	32	6
Induced pluripotent stem cell (GSE25970)	12	6
Re-epithelizing skin cells (GSE28914)	11	8

*Note*: *represents a case where all the cancer samples of the cancer type in TCGA are used instead those with predicted high purity since some of the programs do not apply to the data format of the cancer type.

The expression levels of all genes in all cancer samples of each type form a bimodal distribution (Figure S6), where the first peak (from left to right) represents genes that are not expressed and the second peak is for the expressed genes. For each cancer type, genes with expression values lower than or equal to the lowest point of the valley between the two peaks is considered as *not* expressed. The same criteria apply to the controls as well as the NPC samples from GEO.

### Selection of pH-related transporter genes

We examined all the genes in the transporter families, including bicarbonate transporters, sodium-proton exchangers, anion exchangers, V-ATPase, lactic acid transporters, Ca^2+^-ATPase, and K^+^/H^+^ ATPase. A few transporters localized in plasma membrane were considered as reliable acid-loading or acid-extruding transporters. Details follow.

The family of bicarbonate-transporter genes consists of eleven *SLC4* genes. *SLC4A1*, *2*, *3* are known to exchange extracellular Cl^−^ for intracellular HCO^−^ [52], hence serving as acid loaders; and *SLC4A4-10* tend to cotransport extracellular Na^+^ and HCO_3_^−^ into cells [53], hence acid extruders. It is known that *SLC4A11* does not transport bicarbonate, hence not considered; and *SLC4A6* has not been identified yet. Out of the remaining ones, *SLC4A8* and *A10* are not expressed in any samples, cancer or control. *SLC4A1* and *SLC4A5* are each expressed in only one tissue type: *4A1* in kidney and *4A5* in thyroid. *SLC4A7* tends to be located in focal adhesion sites, in addition to plasma membrane, making their interpretation challenging. Hence, we do not consider any of these *SLC4* transporters, which leaves *SLC4A1AP* (an adaptor protein of *SLC4A1*), *A2*, *A3* as acid loaders, and *SLC4A4* and *A9* as acid extruders.

The family of sodium-proton exchangers consists of nine *SLC9A* genes, which generally exchange extracellular Na^+^ for intracellular H^+^ (or NH_4_^+^), hence acid extruders [54]. The following five *SLC9A* genes are not considered for different reasons in our study. *SLC9A1* and *A7* can be localized to at least three subcellular compartments [55]. *SLC9A8* is localized only in Golgi. *SLC9A5* is not expressed in any samples studied here. *SLC9A6* is predominantly expressed in endosome [56]. This leaves *SLC9A2*, *3*, *4*, *9* for further analyses.

Among the *SLC26* anion exchangers, only six may exchange Cl^−^ for HCO_3_^−^, namely *SLC26A3*–4, 6–7, 9, 11 [57]. *SLC26A3* and *A4* are not expressed in majority of the tissue types under study, hence not considered. *SLC26A7* is mainly a chloride channel, which exchanges Cl^−^ for a range of intracellular anions [58], hence not considered. *SLC26A11* can be localized to five subcellular compartments, hence too non-specific and not considered. This leaves two genes in our study: *SLC26A6* and *A9*. Interestingly, while both transporters exchange Cl^−^ for an anion, including HCO_3_^−^, they facilitate Cl^−^ flux in the opposite directions, namely *SLC26A6* moving extracellular Cl^−^ into cells [59] and *A9* moving intracellular Cl^−^ out [60].

The *ATP6V* genes encoding the V-ATPase complex tend to have multiple subcellular locations. Considering that only those localized to plasma membrane are relevant to our study, we have developed a computational method to de-convolute the observed expressions of the *ATP6V* genes to tease out the portions of these genes whose protein products are localized in the plasma membrane (see File S1). Table S2 lists the estimated average expression levels of the ATP6V genes whose protein products are localized in plasma membrane across different cell types.

Both lactic acid transporter genes, *SLC16A1* and *A3*, are included in our analyses. Neither the Ca^2+^-ATPase (encoded by the *ATP2B1-4* genes) nor the K^+^/H^+^ ATPase (encoded by the *ATP4A*, *B* genes) is particularly informative, hence we did not include them in our analyses.

### Calculation of correlation and statistical significance

To calculate the correlation between the expression of a given gene *g* and a set of *M* genes in a sample set, we choose the first two principle components (PCs) to represent the gene set if they can explain at least 75% of the data variance. A linear regression model was constructed as shown below.

(1) $$e\left(g\right)\;{\beta_{1}PC}_{1}+{\beta_{2}PC}_{2}+\beta_{0}$$

where$e\left(g\right)$ is the expression of gene g; ${\mathrm{PC}}_{1}$ and ${\mathrm{PC}}_{2}$ are the first and second PCs of the expression of the gene set in the given samples; and {$\beta_{i}\}$ are regression parameters. If ${\mathrm{PC}}_{1}$ and ${\mathrm{PC}}_{2}$ fail to explain at least 75% of the data variance, we use the following procedure to assess the statistical significance of the detected correlation between the gene *g* and the set of *M* genes. First, we calculate the Pearson correlations between *g* and each of the *N* genes in the human genome (*N* = 20,000) as the background; and then we conduct a multiple-hypothesis test using the false discovery rate control, to select *n* genes from *N* to achieve statistical significance for each correlation with *g* (*P* < 0.01). The *P* value for the correlation is calculated as:

(2) P=1-∑i=0m-1MiN-Mn-iNn

where *m* is the number of genes in the union of the *n*- and *M*- gene sets. For two gene sets GS_1_ and GS_2_ and their gene expression matrices *M*_1_ and *M*_2_ (across samples), we first calculate their principal curves [61] to capture the nonlinear variance within matrices *M*_1_ and *M*_2_. We then project each sample of M_1_ or M_2_ onto the corresponding principal curve, denoted as DP_1_ and DP_2_, respectively. Pearson’s correlation coefficient (PCC) between DP_1_ and DP_2_ is then calculated to indicate the correlation between the two gene sets and the relevant *p*-value. R package “pathifier” was applied to calculate the principal curve of the gene expression matrix.

### Identification of differentially-expressed genes

We have applied Wilcoxon signed-rank test for cancer samples *vs.* matching controls and NPCs *vs.* controls to identify differentially- expressed genes (DEGs). A gene is considered to be significantly differentially expressed if the difference in its expression is at least 1.3-fold between cancer (or NPCs) and their relevant control samples (FC > 1.3), with the false discovery rate <0.01.

## Authors’ contributions

YX, HS, and YL designed the project; HS, LC, and SC performed the study; HS and LC analyzed the data; YX and HS wrote the paper. All authors read and approved the final manuscript.

## Competing interests

The authors declare no potential conflicts of interest.

## Supplementary material

Supplementary Figure S4 **Time-specific expression data of key genes involved in Warburg effect, electron transport chain and nucleotide synthesis pathways and expression level of *PRKAA* in GSE60234** Gene expression levels of *PKM*, *SLC16A1*(Warburg effect), *COX7A1* (electron transport chain), *PPAT*, *GART* (nucleotide synthesis), and expression level of *PRKAA* where the time course data with 8 time points are grouped into 4 segments with each containing 2 consecutive points and *T_i_* represents the *i*th segment, 0 ≤ *i* ≤ 4.

Supplementary Figure S5 **Percentages of cancer samples in different stages A.** Before purity-based selection. **B.** After purity-based selection. The purity-based selection was not performed for ESCA and STAD, due to the incompatibility of the data formats.

Supplementary Figure S6 **Gene expression density distributions for each cancer type and each NPC type A.** Expression density distributions for each cancer type, one for cancer samples and one for controls. **B.** Expression density distributions for each NPC type, one for activated samples and one for controls. RNA-seq data for ESCA and STAD were generated using Illumina HiSeq v1, and all other cancer data examined here were generated using Illumina HiSeq v2.
