# Supplementary material for: Warburg Effects in Cancer and Normal Proliferating Cells: Two Tales of the Same Name
Source: Genomics Proteomics Bioinformatics. 2019 May 7;17(3):273–86. doi: 10.1016/j.gpb.2018.12.006 (PMC6818181; doi:10.1016/j.gpb.2018.12.006)
Supplement: Supplementary Figure S4 — Time-specific expression data of key genes involved in Warburg effect, electron transport chain and nucleotide synthesis pathways and expression level of PRKAA in GSE60234 Gene expression levels of PKM, SLC16A1(Warburg effect), COX7A1 (electron transport chain), PPAT, GART (nucleotide synthesis), and expression level of PRKAA where the time course data with 8 time points are grouped into 4 segments with each containing 2 consecutive points and Ti represents the ith segment, 0 ≤ i ≤ 4. [file mmc1.ppt]

## Slide 1
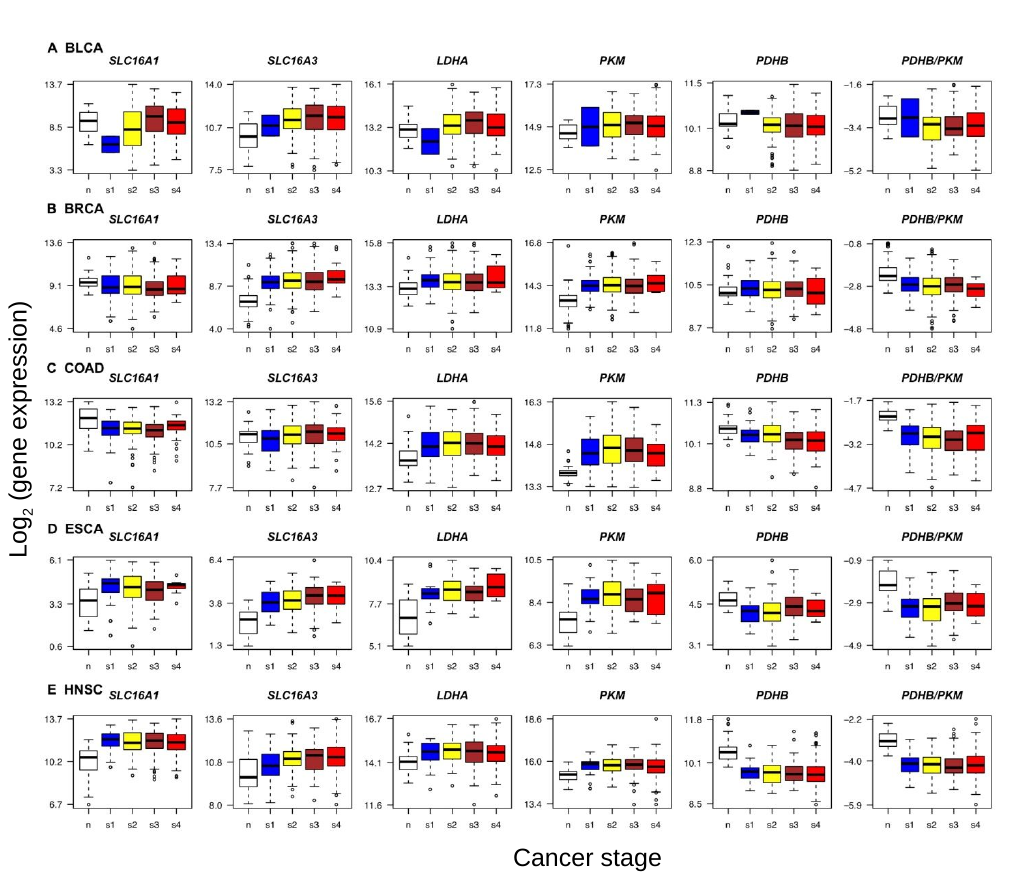

Log2 (gene expression)
Cancer stage

## Slide 2
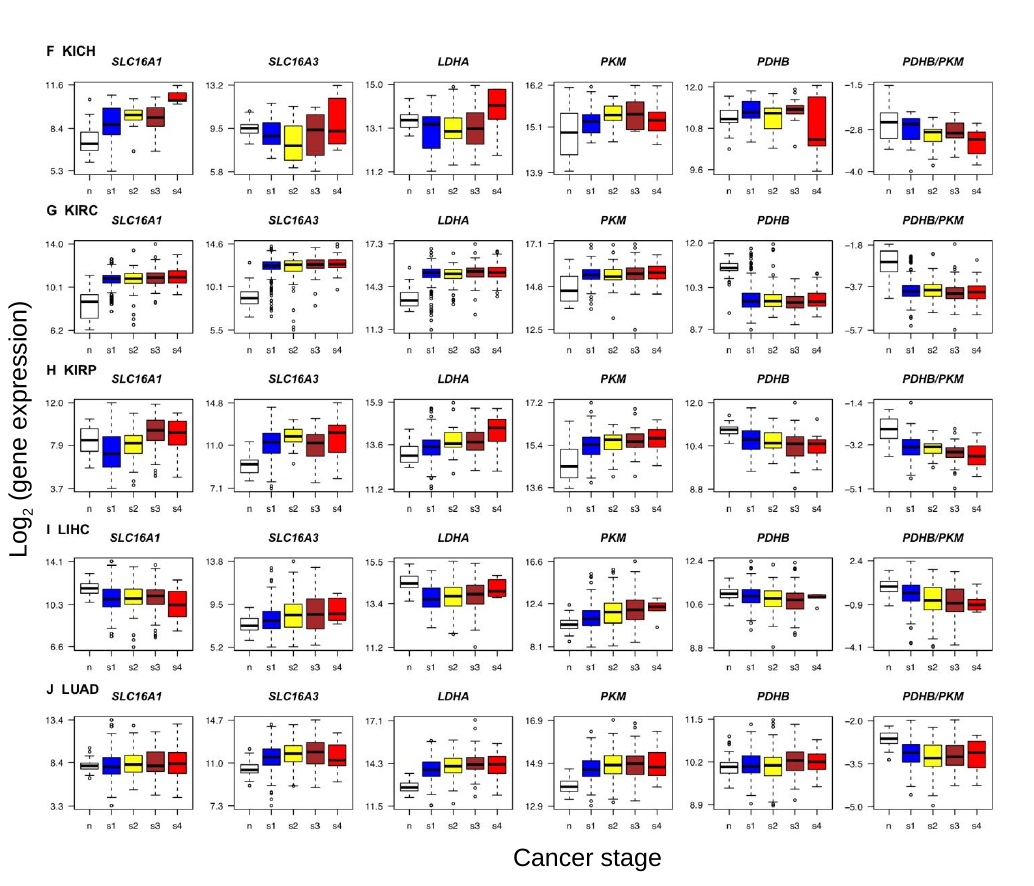

Log2 (gene expression)
Cancer stage

## Slide 3
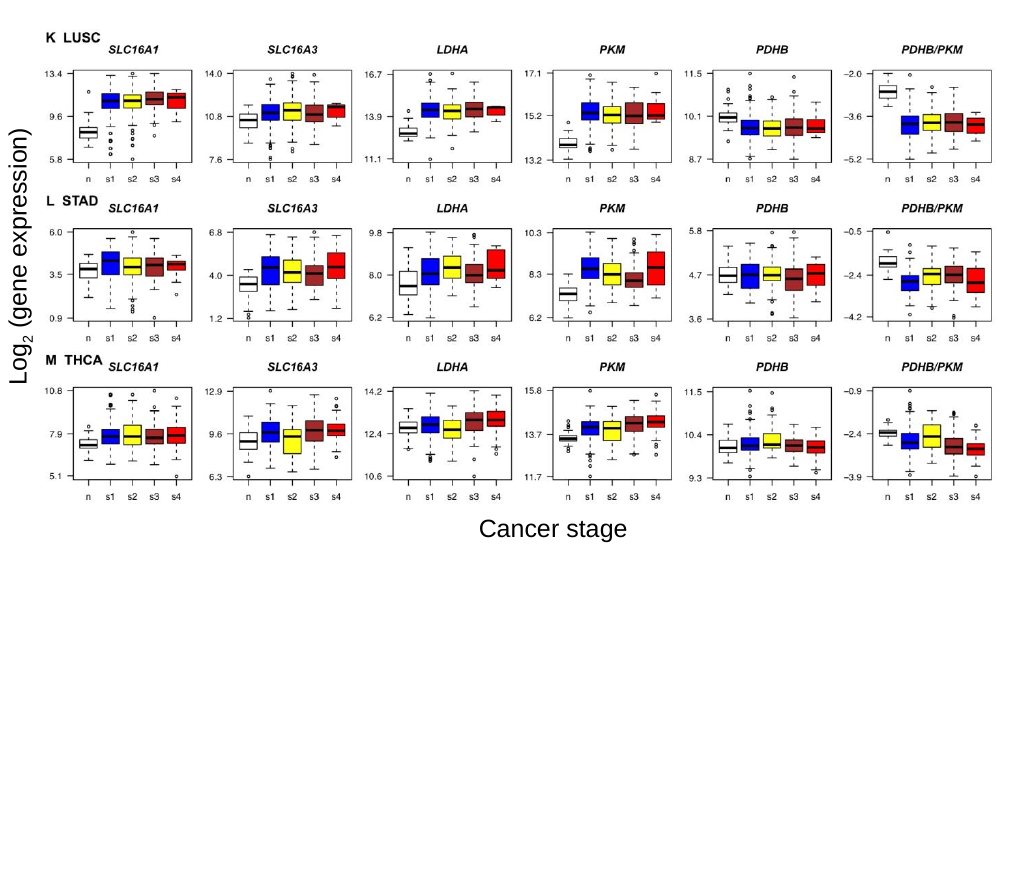

A
Log2 (gene expression)
Cancer stage
Group
