# Supplementary figures and images for: Warburg Effects in Cancer and Normal Proliferating Cells: Two Tales of the Same Name
Source: Genomics Proteomics Bioinformatics. 2019 May 7;17(3):273–86. doi: 10.1016/j.gpb.2018.12.006 (PMC6818181; doi:10.1016/j.gpb.2018.12.006)

## Slide 1
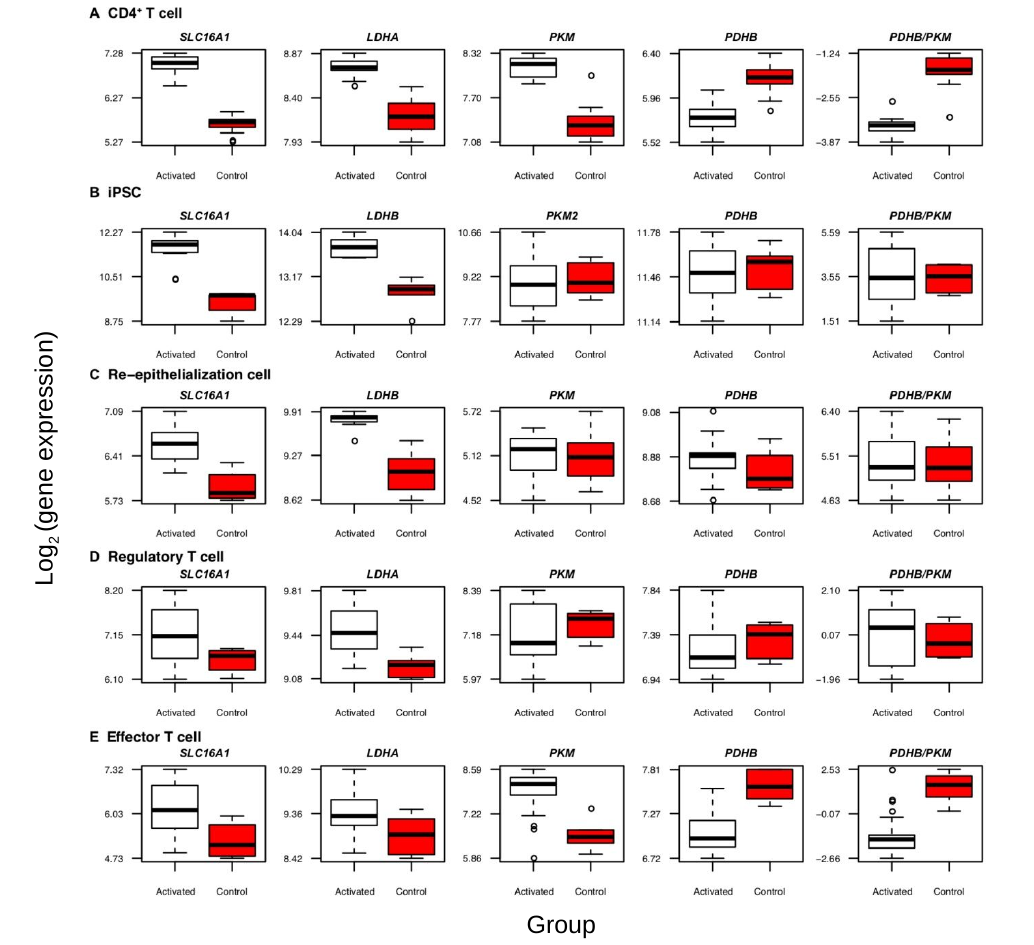

Log2 (gene expression)
Group

Supplement: Supplementary Figure S5 — Percentages of cancer samples in different stages A. Before purity-based selection. B. After purity-based selection. The purity-based selection was not performed for ESCA and STAD, due to the incompatibility of the data formats. [file mmc2.ppt]

## Slide 1
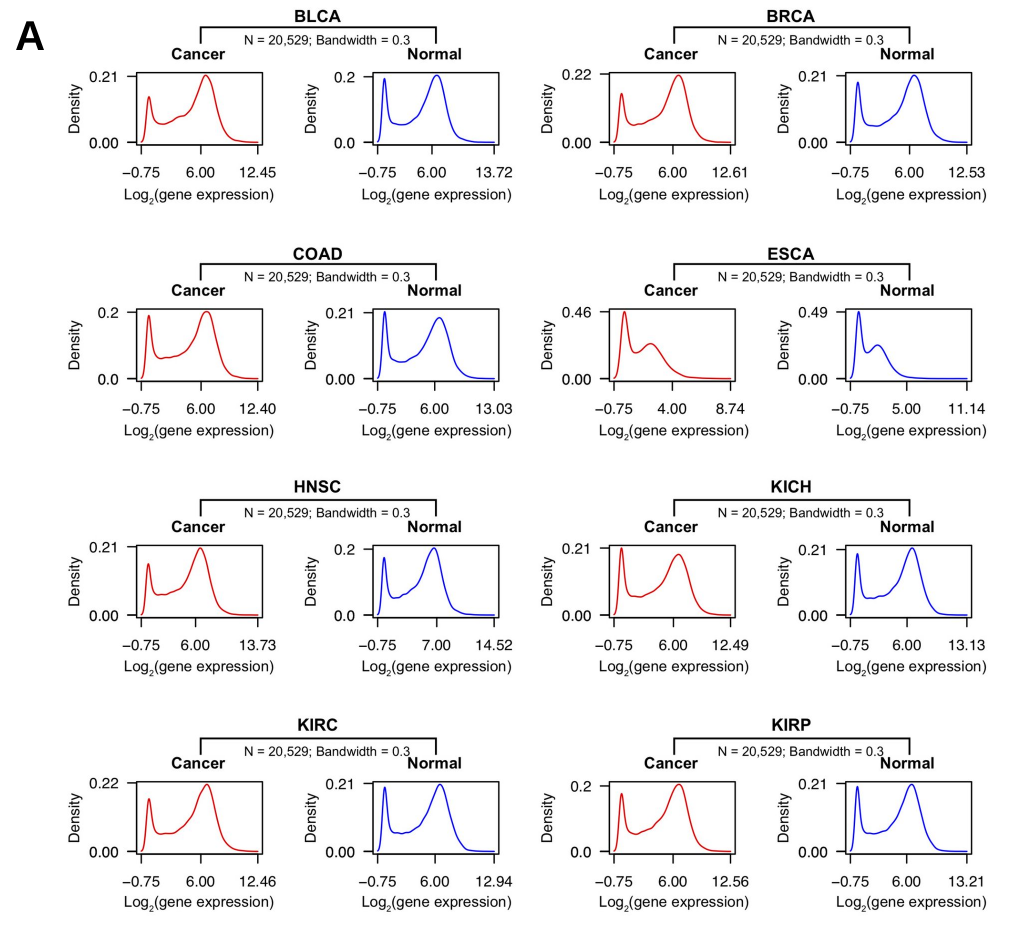

A

## Slide 2
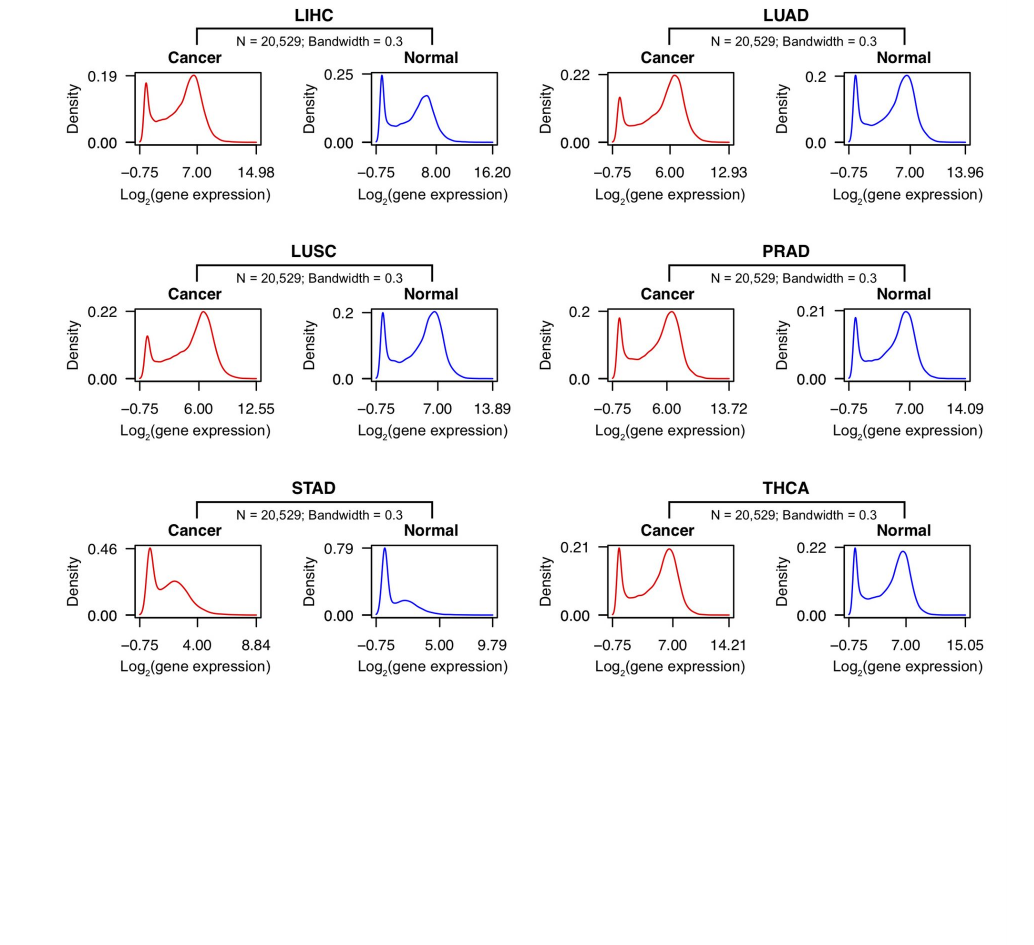

A

## Slide 3
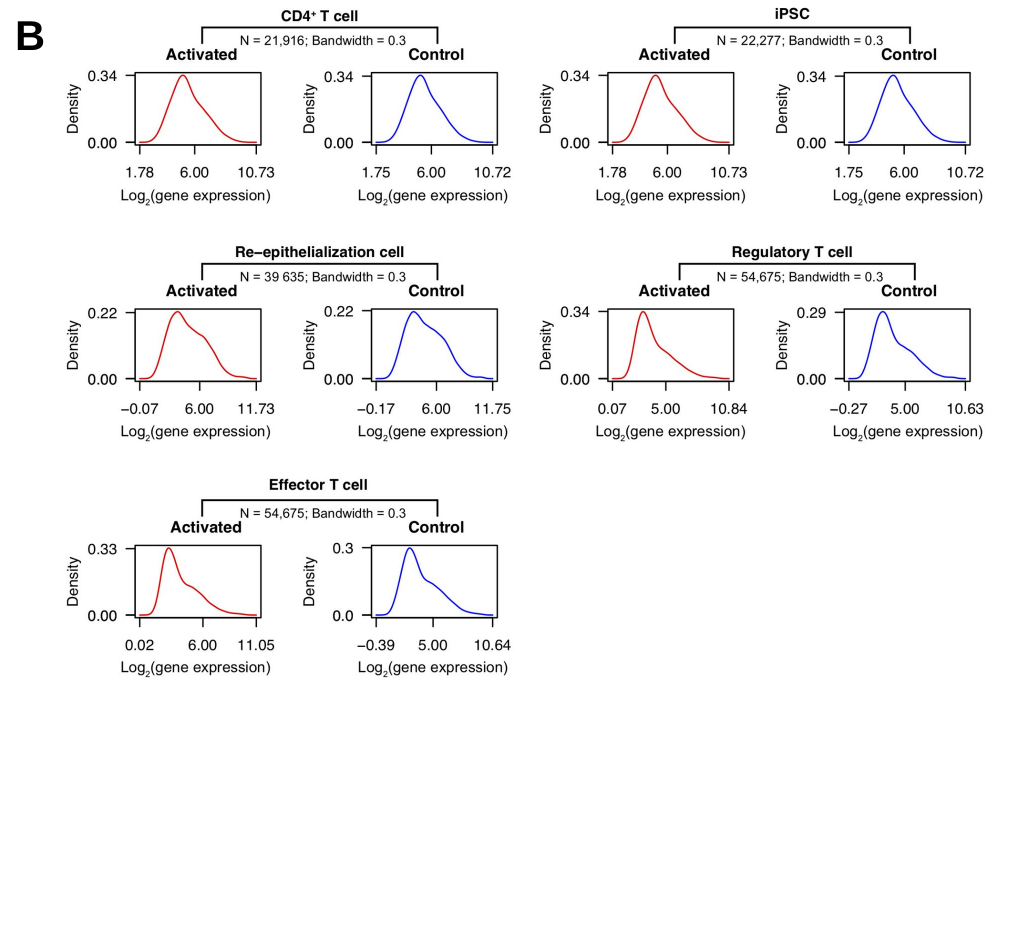

A
B
B

Supplement: Supplementary Figure S6 — Gene expression density distributions for each cancer type and each NPC type A. Expression density distributions for each cancer type, one for cancer samples and one for controls. B. Expression density distributions for each NPC type, one for activated samples and one for controls. RNA-seq data for ESCA and STAD were generated using Illumina HiSeq v1, and all other cancer data examined here were generated using Illumina HiSeq v2. [file mmc3.ppt]
