# Supplementary material for: Warburg Effects in Cancer and Normal Proliferating Cells: Two Tales of the Same Name
Source: Genomics Proteomics Bioinformatics. 2019 May 7;17(3):273–86. doi: 10.1016/j.gpb.2018.12.006 (PMC6818181; doi:10.1016/j.gpb.2018.12.006)
Supplement: Supplementary Table S1 [file mmc4.docx]

**Table S1 Subcellular locations for the *ATP6V* genes**

| \| **Gene name** \| \| **Subcellular locations (confidence score 4 or 5)** \| \| \|  \| **Additional locations (score 3)** \| \| \| \| --- \| --- \| --- \| --- \| --- \| --- \| --- \| --- \| --- \| \| *ATP6V0A1* \| Plasma membrane, extracellular space, nucleus, cytosol, lysosome endosome \| \| \| \| \|  \|  \| \| *ATP6V0A2* \| Plasma membrane, lysosome, endosome \| \| \| \|  \| Cytosol \|  \| \| *ATP6V0A4* \| Plasma membrane, extracellular space, lysosome, endosome \| \| \| \| \| Cytosol \|  \| \| *ATP6V0B* \| Endosome \| \|  \|  \|  \| Plasma membrane \|  \| \| *ATP6V0C* \| Plasma membrane, extracellular space, lysosome, endosome \| \| \| \| \|  \|  \| \| *ATP6V0D1* \| Extracellular space, lysosome, endosome \| \| \| \|  \| Cytosol \|  \| \| *ATP6V0D2* \| Plasma membrane, extracellular space, lysosome, endosome \| \| \| \| \| Cytosol \|  \| \| *ATP6V0E1* \| Endosome \| \|  \|  \|  \| Extracellular space \|  \| \| *ATP6V0E2* \| Endosome \| \|  \|  \|  \|  \|  \| |
| --- | --- | --- | --- | --- | --- | --- | --- | --- | --- | --- | --- | --- | --- | --- | --- | --- | --- | --- | --- | --- | --- | --- | --- | --- | --- | --- | --- | --- | --- | --- | --- | --- | --- | --- | --- | --- | --- | --- | --- | --- | --- | --- | --- | --- | --- | --- | --- | --- | --- | --- | --- | --- | --- | --- | --- | --- | --- | --- | --- | --- | --- | --- | --- | --- | --- | --- | --- | --- | --- | --- | --- | --- | --- | --- | --- | --- | --- | --- | --- | --- | --- |

*Note*: Subcellular locations predicted with confidence scores 4 or 5 are listed in the second column and additional ones, if any, with score 3 are listed in the third column.
