# Supplementary material for: Warburg Effects in Cancer and Normal Proliferating Cells: Two Tales of the Same Name
Source: Genomics Proteomics Bioinformatics. 2019 May 7;17(3):273–86. doi: 10.1016/j.gpb.2018.12.006 (PMC6818181; doi:10.1016/j.gpb.2018.12.006)
Supplement: Supplementary Table S2 [file mmc5.docx]

**Table S2 Average expression levels of the *ATP6V0B* gene contributed by plasma membrane**

| **Dataset** | **Type** | **Control** | **Stage1** | **Stage2** | **Stage3** | **Stage4** |
| --- | --- | --- | --- | --- | --- | --- |
| **Cancer** | **BLCA** | 1048.7 | 2249.8 | 2171.9 | 2441.3 | 2166.7 |
| **Cancer** | **BRCA** | 380.6 | 1281.9 | 1363.1 | 1198.6 | 1222.3 |
| **Cancer** | **COAD** | 1881.6 | **1673.9** | **1840.5** | **1635.7** | **1760.6** |
| **Cancer** | **ESCA** | 23.3 | 33.2 | 31.6 | 34.5 | 38.9 |
| **Cancer** | **HNSC** | 1588.6 | 2011.3 | 2227.0 | 2369.6 | 2410.9 |
| **Cancer** | **KICH** | 2865.0 | 4990.6 | 5236.3 | 4587.8 | 5272.2 |
| **Cancer** | **KIRC** | 1556.7 | **1125.9** | **1172.9** | **1192.0** | **1312.8** |
| **Cancer** | **KIRP** | 1488.1 | **678.8** | **817.7** | **460.4** | **1037.2** |
| **Cancer** | **LIHC** | 171.0 | 732.9 | 1517.5 | 1269.1 | 806.7 |
| **Cancer** | **LUAD** | 1513.1 | 1616.2 | 1604.5 | 1608.7 | **1486.1** |
| **Cancer** | **LUSC** | 1737.7 | 1898.0 | **1734.2** | 1858.2 | 2874.7 |
| **Cancer** | **STAD** | 30.8 | 39.3 | 36.4 | 31.0 | 35.3 |
| **Cancer** | **THCA** | 0.0 | 974.2 | 289.2 | 793.8 | 924.4 |
|  |  | **Control** | **Activated State** | | | |
| **NPC sets** | **CD4^+^ T cell** (GSE60235) | 130 | 170 | | | |
| **NPC sets** | **iPSC** (GSE25970) | 1700 | 1270 | | | |
| **NPC sets** | **Re-epithelizing cell** (GSE28914) | 430 | 440 | | | |
| **NPC sets** | **Regulatory T cell** (GSE11292) | Not expressed | Not expressed | | | |
| **NPC sets** | **Effector T cell** (GSE11292) | 540 | 860 | | | |

*Note*: All number in bold indicate cancer samples have lower V-ATP expression level than the matching controls.
