# Supplementary material for: Warburg Effects in Cancer and Normal Proliferating Cells: Two Tales of the Same Name
Source: Genomics Proteomics Bioinformatics. 2019 May 7;17(3):273–86. doi: 10.1016/j.gpb.2018.12.006 (PMC6818181; doi:10.1016/j.gpb.2018.12.006)
Supplement: Supplementary Table S4 [file mmc7.docx]

**Table S4 Number of up-regulated proteasome genes in cancer and NPCs**

| **Type** | **Number of up-regulated**  **proteasome genes** |
| --- | --- |
| BLCA | 29 |
| BRCA | 37 |
| COAD | 26 |
| ESCA | 34 |
| HNSC | 32 |
| KICH | 10 |
| KIRC | 24 |
| KIRP | 28 |
| LIHC | 27 |
| LUAD | 35 |
| LUSC | 33 |
| PRAD | 21 |
| STAD | 33 |
| THCA | 9 |
| CD4^+^ T cell (GSE60235) | 17 |
| iPSC (GSE25970) | 12 |
| Re-epithelizing cell (GSE28914) | 4 |
| Regulatory T cell (GSE11292) | 0 |
| Effector T cell (GSE11292) | 3 |
