# Supplementary material for: Warburg Effects in Cancer and Normal Proliferating Cells: Two Tales of the Same Name
Source: Genomics Proteomics Bioinformatics. 2019 May 7;17(3):273–86. doi: 10.1016/j.gpb.2018.12.006 (PMC6818181; doi:10.1016/j.gpb.2018.12.006)
Supplement: Supplementary Table S6 [file mmc9.docx]

**Table S6 Genes used to estimate the occurrence of cytosolic Fenton reaction**

| **Functional categories** | **Genes** |
| --- | --- |
| Cytosolic iron sulfur cluster synthesis genes | *ABCB7*, *BRIP1*, *CIAO1*, *CIAPIN1*, *FAM96B*, *MMS19*, *NDOR1*, *NUBP1*, *NUBP2*, *POLD1*, *RTEL1* |
| Cytosolic genes for neutralizing H_2_O_2_ | *HMOX1*, *HMOX2*, *NCF2*, *NCF4*, *NOX1*, *NOX4*, *NOX5*, *NOXA1*, *NOXO1*, *PRDX2*, *PRDX4* |
| Cytosolic NAD(P)H dehydrogenase genes | *ADH1A*, *ADH1B*, *ADH1C*, *ADH4*, *ADH6*, *ADH7*, *AKR1B1*, *AKR1B10*, *AKR1C1*, *AKR1C2*, *AKR1C3*, *AKR1C4*, *ALDH1A1*, *ALDH1A2*, *ALDH1A3*, *ALDH3A1*, *ALDH3B1*, *ALDH3B2*, *ART1*, *ART3*, *ART4*, *ART5*, *BDH2*, *BLVRA*, *BLVRB*, *BST1*, *C5orf4*, *CD38*, *CRYL1*, *CRYZ*, *CYP39A1*, *CYP46A1*, *CYP4F2*, *CYP4F3*, *CYP51A1*, *CYP7A1*, *CYP7B1*, *CYP8B1*, *DHPS*, *DUOX1*, *DUOX2*, *FDFT1*, *FMO1*, *FMO2*, *FMO3*, *FMO4*, *FMO5*, *GAPDH*, *GAPDHS*, *GPD1L*, *GRHPR*, *H6PD*, *HPGD*, *HSD17B1*, *HSD17B12*, *HSD17B2*, *HSD17B6*, *HSD17B7*, *HSD17B8*, *HSD3B7*, *IMPDH1*, *IMPDH2*, *LDHAL6A*, *LDHC*, *MDH2*, *MSMO1*, *MTHFR*, *MTRR*, *NADK*, *NOS2*, *NOS3*, *NQO1*, *NSDHL*, *NUDT12*, *PHGDH*, *PTGR1*, *PTGR2*, *PYCR2*, *PYCRL*, *SC5D*, *SDR16C5*, *SIRT6*, *SLC23A1*, *SLC23A2*, *UGDH*, *XDH* |
| Exogenous superoxide synthesis genes | *SOD1*, *CYBA*, *CYBB*, *NCF2*, *NOX1*, *SOD2*, *VDAC1*, *VDAC2*, *VDAC3* |
| Cytosolic iron transporter genes | *FTL*, *FTH1*, *FTMT*, *FTHL17*, *TF*, *TFRC*, *TFR2*, *STEAP1*, *STEAP2*, *STEAP3*, *STEAP4*, *CYBRD1*, *FRRS1*, *SLC39A14*, *SLC11A2*, *SLC40A1* |
| Proteasome genes | *PSMA1*, *PSMA2*, *PSMA3*, *PSMA4*, *PSMA5*, *PSMA6*, *PSMA7*, *PSMA8*, *PSMB10*, *PSMB11*, *PSMB1*, *PSMB2*, *PSMB3*, *PSMB4*, *PSMB5*, *PSMB6*, *PSMB7*, *PSMB8*, *PSMB9*, *PSMC1*, *PSMC2*, *PSMC3*, *PSMC4*, *PSMC5*, *PSMC6*, *PSMD10*, *PSMD11*, *PSMD12*, *PSMD13*, *PSMD14*, *PSMD1*, *PSMD2*, *PSMD3*, *PSMD4*, *PSMD5*, *PSMD6*, *PSMD7*, *PSMD8*, *PSMD9* |
