# Supplementary material for: Warburg Effects in Cancer and Normal Proliferating Cells: Two Tales of the Same Name
Source: Genomics Proteomics Bioinformatics. 2019 May 7;17(3):273–86. doi: 10.1016/j.gpb.2018.12.006 (PMC6818181; doi:10.1016/j.gpb.2018.12.006)
Supplement: Supplementary Table S9 [file mmc12.docx]

**Table S9 Differential expressions level of the selected cell proliferation and Warburg effect genes in GSE77239**

| **Gene symbol** | **Log_2_FC** |
| --- | --- |
| *CCNA2* | −0.75 |
| *CCNB1* | −0.881 |
| *CCNB2* | −0.555 |
| *CCNB3* | −0.192 |
| *CCND1* | −0.426 |
| *CCNE1* | −0.264 |
| *CCNE2* | −0.828 |
| *CDC20* | −0.493 |
| *CDC23* | −0.66 |
| *CDC25A* | −0.984 |
| *CDC25C* | −0.337 |
| *CDC45* | −0.673 |
| *CDC6* | −0.734 |
| *CDC7* | −0.544 |
| *CDK1* | −1.156 |
| *CDK2* | −0.532 |
| *CDK20* | −0.112 |
| *CDK2AP1* | −0.286 |
| *CDK4* | −0.488 |
| *POLA1* | −0.636 |
| *POLD3* | −0.177 |
| *POLE2* | −0.535 |
| *POLE4* | −0.187 |
| *POLG2* | 0.447 |
| *SLC16A1* | −0.298 |
| *LDHA* | −0.253 |
| *PGK1* | −0.966 |
| *PKM* | −0.184 |
