# Supplementary material for: Warburg Effects in Cancer and Normal Proliferating Cells: Two Tales of the Same Name
Source: Genomics Proteomics Bioinformatics. 2019 May 7;17(3):273–86. doi: 10.1016/j.gpb.2018.12.006 (PMC6818181; doi:10.1016/j.gpb.2018.12.006)
Supplement: Supplementary Table S10 [file mmc13.docx]

**Table S10 Samples of cancer tissues of 14 types in the TCGA database before purity selection**

| **Cancer type** | **No. of cancer samples** | **No. of control samples** |
| --- | --- | --- |
| Bladder urothelial carcinoma (BLCA) | 408 | 19 |
| Breast invasive carcinoma (BRCA) | 1095 | 113 |
| Colon adenocarcinoma (COAD) | 285 | 41 |
| Esophageal carcinoma (ESCA) | 184 | 13 |
| Head and Neck squamous cell carcinoma (HNSC) | 520 | 44 |
| Kidney chromophobe (KICH) | 66 | 25 |
| Kidney renal clear cell carcinoma (KIRC) | 533 | 72 |
| Kidney renal papillary cell carcinoma (KIRP) | 290 | 32 |
| Liver hepatocellular carcinoma (LIHC) | 371 | 50 |
| Lung adenocarcinoma (LUAD) | 515 | 59 |
| Lung squamous cell carcinoma (LUSC) | 501 | 51 |
| Prostate adenocarcinoma (PRAD) | 497 | 52 |
| Stomach adenocarcinoma (STAD) | 238 | 33 |
| Thyroid carcinoma (THCA) | 505 | 59 |
